# A Strategic Study about Quality Characteristics in e-Health Systems Based on a Systematic Literature Review

**DOI:** 10.1155/2015/863591

**Published:** 2015-06-04

**Authors:** F. J. Domínguez-Mayo, M. J. Escalona, M. Mejías, G. Aragón, J. A. García-García, J. Torres, J. G. Enríquez

**Affiliations:** Web Engineering and Early Testing (IWT2) Research Group, Department of Computer Languages and Systems, University of Seville, Avenida Reina Mercedes s/n, 41012 Seville, Spain

## Abstract

e-Health Systems quality management is an expensive and hard process that entails performing several tasks such as analysis, evaluation, and quality control. Furthermore, the development of an e-Health System involves great responsibility since people's health and quality of life depend on the system and services offered. The focus of the following study is to identify the gap in Quality Characteristics for e-Health Systems, by detecting not only which are the most studied, but also which are the most used Quality Characteristics these Systems include. A strategic study is driven in this paper by a Systematic Literature Review so as to identify Quality Characteristics in e-Health. Such study makes information and communication technology organizations reflect and act strategically to manage quality in e-Health Systems efficiently and effectively. As a result, this paper proposes the bases of a Quality Model and focuses on a set of Quality Characteristics to enable e-Health Systems quality management. Thus, we can conclude that this paper contributes to implementing knowledge with regard to the mission and view of e-Health (Systems) quality management and helps understand how current researches evaluate quality in e-Health Systems.

## 1. Introduction

As far as e-Health definition is concerned, Eysenbach [1] defines it as “an emerging field in the intersection of medical informatics, public health and business, referring to Health services and information delivered or enhanced through the Internet and other related technologies.” In a broader sense, the term characterizes not only a technical development, but also a state of mind, a way of thinking, an attitude, and a commitment for networked information and global thinking, so that information and communication technology can improve healthcare locally, regionally, and worldwide. e-Health Systems provide the umbrella framework to describe both the comprehensive management of Health information through computerized systems and the safe exchange among consumers, providers, the government, and quality entities and insurance companies. In general, e-Health is increasingly considered to be the most promising tool for improving the overall quality, safety, and efficiency of the Health delivery system [2]. ICTs (Information and Communication Technologies) can have a massive impact on all aspects of healthcare, ranging from providing people with the information they need to live a healthy lifestyle to supplying new tools available for designing the future of medicine. This way, ICTs guarantee more efficient and responsive healthcare systems addressed to patients, by offering mobile Health technologies and most importantly “at home” technologies. e-Health is, therefore, an emerging and important new global industry. It is not simply that e-Health Systems were replaced by electronic cards, but ICTs also enable better personalized care. This is to make treatments more effective, to entrust doctors to diagnose problems faster, and even to foresee problems before they appear. ICTs can also improve healthcare more directly allowing patients to be examined in real time, either from home or during transportation, improving their lifestyle and playing a key role in numerous processes.

As far as e-Health Systems quality evaluation work is concerned, Goletsis and Chletsos [3] have the intention of providing such a systemic approach for the multidimensional assessment of e-Health/telemedicine interventions. They offer a set of dimensions to be combined in order to test e-Health applications based on the needs of the multiple stakeholder healthcare environments which include technical quality, medical completeness, effectiveness, usability, organizational fit, and cost, as a few results of the evaluation. Mettler and Vimarlund [4] discuss in this paper a first attempt on how to put the value of the planned Health Systems changes into practice. Generic allocation decisions related to a utility and a readiness portfolio are formulated to give priority to investments as well as identify weak points in the defined e-Health strategy. Pagliari [5], on his part, offers a personal viewpoint based on a nonsystematic review of the literature and the experience of observing and participating in the design, evaluation, and analysis of Health informatics interventions. Pagliari discusses the importance of doing research with the aim of ensuring that new e-Health technologies are adopted effectively.

By the same token, Greenhalgh and Russell [6] offer an alternative set of guiding principles for e-Health evaluation based on traditions that view evaluation as a social practice rather than a scientific testing and illustrate it using England's controversial Summary Care Record program as an example. In addition, Eysenbach [1] suggest an e-Health System characterization in terms of the issues addressed in the articles published by the Journal of Medical Internet Research. The elements involved in such characterization are efficiency, enhancement of quality of care based on evidence, empowerment of consumers and patients, encouragement of a new patient-professional relationship, education of physicians through online sources, consumers, possibility of information exchange and communication in a standardized way, expansion of the scope of healthcare beyond its conventional boundaries and ethics, and equity of the e-Health System.

ISO 27799:2008 [7] defines the guidelines to support the interpretation and implementation of Health informatics in ISO/IEC 27002 [8] and it is a companion to that standard. ISO 27799:2008 specifies a set of detailed controls for managing Health information security and provides Health information security best practice guidelines. Standard provides guidance to healthcare organizations and other personal Health information custodians on how to best protect the confidentiality, integrity, and availability of such information by implementing ISO/IEC 27002.

ICT service management which encompasses ITSM (IT service management) or ICT services as such [7] is a discipline dealing with managing information technology (ICT) systems, philosophically focused on the customer's perspective of ICT's contribution to business. ITSM stands in deliberate contrast to technology-centered e-Health Systems, ICT management, and business interaction. ITSM is process-focused and, in this sense, it has ties and common interests in process improvement movement (e.g., CMMI-SVC [9], ITIL [10], and ISO 20000 [11]) frameworks and methodologies.

ITIL [7] advocates that ICT services must be aligned with the needs of the business and underpins the core business processes. It advises organizations on how to use ICT as a tool to facilitate business change, transformation, and growth. ITIL best practices are currently detailed within five core publications that make available a systematic and professional approach to manage ICT services by enabling organizations to deliver appropriate services and constantly ensuring that they are meeting business goals and delivering benefits. For this reason, ISO 20000 [11] standard was conceived to fill this gap. Initiated by the two organizations itSMF (IT Service Management Forum) and BSI (British Standard Institute), it is modeled upon the principles of ITIL and, for the first time, it provides ICT organizations with the possibility of certifying their ICT Service Management. Unlike ITIL books, ISO 20000 does not particularly recommend how to design processes. It rather consists of a set of requirements to meet in order to be qualified to obtain ISO 20000 certification. ISO 20000 is based on ITIL V.2 and current ITIL version is V.3.

The main goal of this study is to establish a set of Properties and Quality Characteristics to manage quality in different e-Health Systems under some specific criteria. The paper is organized into the following sections. After this introduction and general analysis of the situation, Section 2 examines the strategic study, while, based on such study, it also lays the foundation for e-Health Systems stakeholders' value and presents the results obtained. In addition, Section 3 introduces a framework that helps managing quality in e-Health Systems efficiently. Concluding the paper is Section 4 by stating conclusions, contributions, and possible future work.

## 2. A Strategic Study for e-Health Systems

The objective of this strategic study is to assist in how to design, develop, and implement e-Health Systems. It tends to ensure that ICT organizations, which are responsible for implementing these systems, attain operational effectiveness and supply distinctive services to the e-Health Systems stakeholder. Its ultimate goal is to make the ICT organization think and act in a strategic manner.

An e-Health System is figured out in the present work as a relatively recent term denoting healthcare practice supported by electronic processes and communication. However, it can also coin different meanings: some people argue that it is exchanged with Health informatics with a broader definition covering electronic/digital processes in Health, while others use it in the narrower sense of healthcare practice by means of the Internet.

This way, ICT organizations of e-Health Systems have to meet stakeholders' needs. Nonetheless, ICT organizations must guarantee stakeholders that, despite these Properties, a set of Quality Characteristics such as Usability, Functionality, or Maintainability must also be covered. To sum up, it is worth stating that the real value of a system depends on these two factors: Properties and Quality Characteristics.

### 2.1. The Systematic Literature Review for Quality Characteristics

There are many reasons for conducting a Systematic Literature Review (SLR). The most common ones for this study focus on the identification of gaps in e-Health Systems quality management in order to suggest areas for further investigation and to provide a background, so that new research activities related to e-Health quality issues must be appropriately set. Specifically, we aim to determine the most studied Quality Characteristics in e-Health Systems so as to identify any gap in this area.

Due to the fact that defining Quality Characteristics involves a large initial effort, the description domain in our approach is identified through a SLR and the review method is based on the research protocol. This section defines the search strategy, sources, studies selection, and selection execution.

#### 2.1.1. Methodology

The guidelines for the systematic review stated in this work follow the protocol defined by Kitchenham [12, 13], which is one of the most acknowledged in software engineering. In addition, we take into consideration the conclusions of Wohlin and Prikladnicki [14] about SLRs in software engineering, who consider that the search strategy is key to ensuring a good starting point for the identification of studies and ultimately for the actual outcome of a particular study. Nevertheless, this proposal initially centers on the systematic reviews of research studies. For this aim, we have adapted this proposal to focus on studies of the state of the art of e-Health Systems and all those related fields. A SLR essentially involves three phases: (i) planning the review, (ii) conducting the review, and (iii) reporting the review.

#### 2.1.2. Planning the Review

The focus of this paper is detecting not only which are the most studied, but also which are the most used Quality Characteristics of e-Health Systems. A review protocol, which is specified in Table 1, specifies the methods that will be used to undertake a specific systematic review. It summarizes the review protocol of the SLR. The authors of the present work do all agree on the review protocol at the moment of conducting the review.

The review protocol is a critical element in any systematic review. All authors review and criticize the protocol in order to find out the appropriate one. In addition, as Kitchenham et al. [12, 13] recommend, this protocol is checked to confirm that

- the search strings are appropriately derived from the research questions;
- the data to be extracted will properly address the research questions;
- the data analysis procedure is appropriate to answer the research questions.

#### 2.1.3. Conducting the Review

Once all planning phase goals have been achieved, the review process leads to the review phase itself, which consists in finding and evaluating whether many primary studies associated with the research questions are adequate and relevant enough to be possible sources for further analysis. The primary studies are searched in the aforementioned databases by means of the keywords represented in Table 1. After carrying out the search, a strategic definition for evaluating the adequacy and relevance of the studies is needed.

Firstly, a search with keywords of Table 1 is run for each logical criterion in the search field. Secondly, the set with the previous primary studies is reduced according to the following inclusion criteria:

- The primary study must have been published from 2006. In the context of this work, this exclusion criterion is considered realistic and acceptable. Therefore, the considered primary studies must be within these last years in order to infer practical conclusions.
- The paper must focus on the quality evaluation of e-Health Systems.
- The paper must have been published in any international journal or conference proceedings, book, or book chapter of interest.

This section aims to choose the sources to complete searches for primary studies. First of all, we carried out preliminary searches with the intention of both identifying existing systematic reviews and assessing the volume of potentially relevant studies, so that in this case we found the following relevant initiatives: Asoh and Rivers [15], Bangert et al. [16], Esteves [17], Ferrer-Roca et al. [18], Gama et al. [19], Golemanov et al. [20], Goletsis and Chletsos [3], Gutiérrez and Riveill [21], Harris et al. [22], Holbrook et al. [23], Hadwich et al. [24], Kastania and Kossida [25], Liu and Park [26], Monda et al. [27], Moumtzoglou and Kastania [28], Palos et al. [29], Pate and Turner-Ferrier [30], Ruxwana et al. [31], Ferrer-Roca et al. [18], Smedberg [32], Tan et al. [33], and Zvikhachevskaya et al. [34]. Secondly, a Web-search was performed to find out other relevant and new concepts associated with our Quality Characteristics. Several sets of keywords were used by combining the concepts of our study, such as “Quality” AND “e-Health”, “Evaluation” AND “e-Health”, “Assessment” AND “e-Health”, “Technology” AND “Health”, or “Communication” AND “Health”. To conclude, our last step consisted in looking for references of papers in the previous reviews in order to identify more concepts.

Thirdly, a new selection is discarded by means of a fast reading of each primary study. First of all, the title theme of the primary study must be linked to the topic of this work. Once this condition is satisfied and cataloguing this primary study as promising, the introduction and abstract must mention the goals of the research question posed in this section.

The selection criterion to evaluate study sources will be availability, not only for consulting articles on the Internet or the digital library of the University of Seville, which contains e-books and has access to other resources such as Google Scholar, Scopus, Mendeley, Science Direct, ISI Web of Knowledge, ACM Digital Library, CiteSeerX, or the IEEE digital library, but also for looking for other tools or mechanisms through keywords and literature from companies, books, journals, and conferences, written by experts in the field.

- * Search Type 1.* As there are many works published, our search started gathering those papers that only included concepts in the Title, excluding those issued prior to 2006. Then, we analyzed which of them covered our domain, and all studies dealing with Quality in e-Health Systems were included. If the paper was not related to that topic, then it was excluded from the study.
- The process so as to take into consideration a paper within our research was as follows: firstly, we considered the Title and Abstract, Keywords, Content, and finally Conclusions of the paper. Most papers were included in that type of search, since the concept word in the Title let us know that the paper focused on that Quality Characteristic concept. Besides, when a paper deals with Quality, it refers to a general concept of quality involving a set of Quality Characteristics. Such particular Quality Characteristics were noticed after an in-depth reading of the paper.
- * Search Type 2*. Later, we looked for the concepts included in the Abstract, Keyword, and Title, excluding again those papers issued prior to 2006. Nevertheless, the high number of papers found by search engines constituted a relevant figure of interest. Due to such an unexpected figure, an in-depth reading of all papers was not viable. Therefore, the goal in Search Types 2 and 3 was to reach the max limit in the number of papers where the Quality Characteristic concept was used. This way, we were only interested in the most used Quality Characteristics concepts in e-Health Systems.
- In addition, we had some problems with some search engines, such as Mendeley search engine, which did not work with long-term conditions in this kind of search and was not suitable for Search Types 2 and 3. Looking for Abstract, Keyword, or Title, in Google Scholar search engine, was not possible; thus, only Scopus stood as the eligible search engine for this surf.
- * Search Type 3*. Finally, we entered the concepts in* All fields*. In the same way as Search Type 2, we were only interested in the most used Quality Characteristics concepts in e-Health Systems. All papers found were collected in order to have a max limit of papers including the Quality Characteristic concept. Besides, we faced some problems with some search engines and in that kind of search, Science Direct, each word was written with quotation marks to ensure that it was totally considered in the documents.

Quality management in e-Health Systems can be regarded as an improvement in healthcare quality, prevention from medical errors, reduction of paperwork, reduction of healthcare costs, expansion of access to affordable care, or implementation of administrative efficiencies. On the one hand, Properties describe the environment or context under study or the needs that ICT organizations must satisfy. On the other hand, Quality Characteristics are the quality aspects that ICT organizations have to guarantee users while utilizing all these Properties. So, these Quality Characteristics are influenced by Properties. For instance, an e-Health System provides a set of tools and services that ICT organizations have to cover to stakeholders' Properties. Moreover, ICT organizations have to guarantee stakeholders that these Properties will be covered together with a set of Quality Characteristics of e-Health Systems, such as Interoperability, Security, Usability, or Accessibility. Hence, the Quality Model consists of a set of Properties, Quality Characteristics, and the relationships among them, and basically, it supports the basis for quality management. It may be defined as “conformance to requirements” and/or “fitness of use.” Furthermore, if they focus on their business, ICT organizations can manage e-Health Systems quality efficiently and effectively.

Then, we carried out the procedures to select the studies in order to obtain articles to verify whether the studies fit both the inclusion and the exclusion criteria. Most papers were obtained from Google Scholar, Mendeley, Scopus, and Science Direct. Some consideration for each Search Type is explained below.

- *Search Type 1*. We had to divide some keywords in order to improve the process. For instance, some keywords like “Resource Utilization” or “Time Behavior” had to be separated in “Resource,” “Utilization,” “Time,” and “Behavior” to increase the range of possibilities. Every paper was read to detect the Quality Characteristics analyzed in our paper. Most papers were considered in this type of search since most fit the domain of interest. The ratio of suitable papers was higher than 60%. Thus, these results were given a 60% degree of importance.
- *Search Type 2*. Mendeley search engine did not contemplate long-term conditions in this type of search involving Abstract, Keyword, and Title and was not considered for Search Types 2 and 3. The same happened with Google Scholar search engine to look for the Abstract, Keyword, and Title. Finally, only Scopus was used for this type of search. We were interested in getting a max limit in the use of Quality Characteristics concepts. Then, due to the fact that the Abstract, Keyword, and Title are relevant fields in the paper containing close contents to those of every paper, we consider 30% of importance for these results. This is a very low rate in stark contrast to the importance rate obtained in Search Type 1.
- *Search Type 3*. Finally, in this type of search involving all fields in the papers, we realized that in Science Direct each word was written with quotation marks to ensure that it was totally considered in the documents.

**Figure 1:**
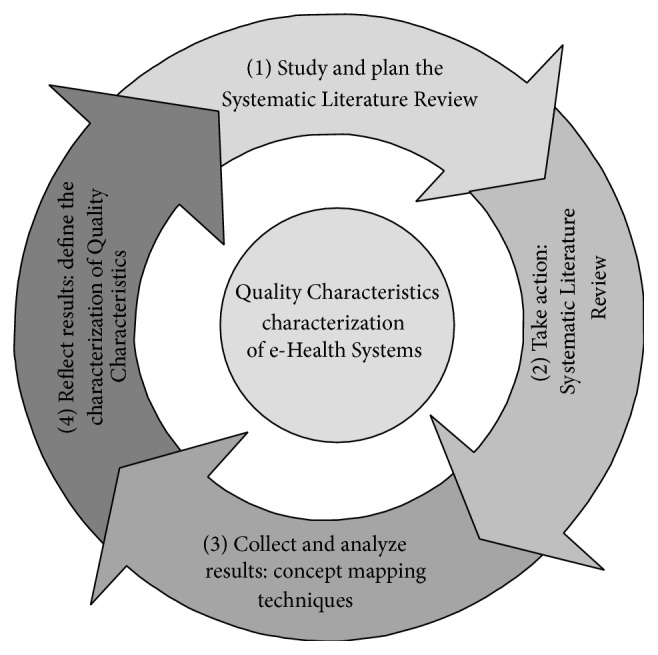
Method to group Quality Characteristics.

In order to group and characterize Quality Characteristics, concept mapping techniques have been carried out. A concept map is a type of graphic organizer used to help people organize and represent knowledge of a subject. Concept maps begin with a main idea (or concept) and then branch out to show how that main idea can be broken down into specific topics. So, a concept map was used to build the characterization of Quality Characteristics during the SLR as shown Figure 1.

This figure shows the process to build the characterization. First of all, we plan the review; then, the review process is carried out. Then, the results are collected and analyzed by the concept mapping techniques and finally, the results are reflected in the characterization of Quality Characteristics. This is a continual cycle of improvements. In consequence, all concepts that were found in the SLR were considered in the concept map to characterize Quality Characteristics. We have also considered ISO 25000 [8] and ITIL [7] because we found that e-Health Systems can be evaluated both as a product and a service.

Concept maps are typically hierarchical, with the subordinate concepts stemming from the main concept or idea. This type of graphic organizer, however, always allows change and new concepts to be added.

- *Start with a main idea, topic, or issue to focus on*. A helpful way to determine the context of your concept map is to choose a focus question.
- *Then determine the key concepts*. Find the key concepts that connect and relate to your main idea and rank them; most generally, inclusive concepts come first and then link to smaller, more specific concepts.
- *Finish by connecting concepts—creating linking phrases and words*. Once the basic links between the concepts are created, add cross-links, which connect concepts in different areas of the map.

#### 2.1.4. Reporting the Review

In this section, we are going to do a specification of dissemination mechanisms and formatting of the main report. It must be mentioned that the Quality Characteristics for e-Health Systems can be grouped in two categories: External/Internal Quality and Quality in Use, the latter depending on the former.

*External/Internal Quality.* It measures e-Health Systems and their behaviour. These are Functionality, Usability, Accessibility, Reliability, Maintainability, and Portability.

- *Functionality*. It is the quality or state of being functional. It specially relates to the set of functions or capabilities associated with computer software, hardware, or any electronic devices.
  - *Security*. It deals with data protection to ensure that only authorized personnel have access to the information.
  - *Interoperability*. It refers to the ability of two or more components or services to exchange information and use that information exchanged.
  - *Accuracy*. It is the condition or quality of being true, correct, or exact; freedom from error or defect; precision or exactness; correctness.
  - *Compliance*. It deals with following standards, regulations, and other requirements.
- *Suitability*. It points out a set of attributes that have influence both on the effort needed for usage and on the individual assessment that a stated or implied set of users make of such usage.
- *Usability*. It is the capability of a feature to be understood, learned, and used when applied under specific conditions.
- *Accessibility*. This general term describes the degree to which a product, device, service, or environment is available for as many people as possible.
- *Reliability*. It is the ability of a system or component to perform its required functions under stated conditions for a specified period of time.
- *Maintainability*. It refers to the ease with which a product can be maintained to isolate and correct defects or their causes like meeting new requirements, making future maintenance easier, or coping with a changed environment.
- *Continuity*. It means the ability a feature has to reach the state or quality of being continuous.
- *Efficiency*. It describes the extent to which time or effort is well used for the intended task or purpose. It is often used with the specific purpose of relying on the capability of a feature to produce a specific outcome effectively with a minimum amount or quantity of waste, expense, or unnecessary effort.
- *Portability*. It is the use of the same system in different environments or platforms. Portability is the key issue for reducing cost, whenever systems with the same functionality are produced for several computing platforms.

*Quality in Use*. It measures the effect of using the e-Health System in a specific context: Safety, Effectiveness, Satisfaction, and Productivity are the state of the e-Health System. They are briefly defined as follows:

- *Safety*. It refers to the state of being “safe,” the condition of being protected against physical, social, spiritual, financial, political, emotional, occupational, psychological, educational, or other types of aspects or consequences of failure, damage, error, accidents, harm, or any other event which could be considered nondesirable for the system.
- *Effectiveness*. It is the capability of producing a desired result. Something is deemed effective, when it has either an intended or expected outcome or produces a deep or vivid impression.
- *Satisfaction*. It associates with different situations such as the act of satisfying or the state of being satisfied; gratification of desire; contentment in possession and enjoyment; and the repose of mind resulting from compliance with desires or demands.
- *Productivity*. It measures production efficiency. Productivity is a ratio of production output which points out what is required to be produced (inputs). Productivity measure is defined as a total output per unit of a total input.

On the contrary, as far as features of Properties definition are concerned, e-Health Systems can encompass a range of services or systems that are at the edge of medicine/healthcare and information technology, including the following.

- *Telemedicine*. It is related to the distance physical and psychological treatments.
- *M-Health*. It includes the use of mobile devices when collecting aggregate and patient level Health data, providing healthcare information to practitioners, researchers, and patients, real-time monitoring of patient vitals, and direct provision of care (via mobile telemedicine).
- *Electronic Health Records (EHR)*. It evolves the concept defined as a systematic collection of electronic Health information about individual patients or population. It is an online record that can be theoretically shared across different healthcare settings.
- *Healthcare Information Systems (HIS)*. It refers to software solutions for appointment scheduling, patient data management, work schedule management, and other administrative tasks involving Health.
- *Consumer Health Informatics (CHI)*. It copes with the use that healthy individuals or patients make of electronic resources dealing with medical topics.
- *Health Knowledge Management (KM)*. It is linked to healthcare knowledge, for instance, in an overview of latest medical journals, best practice guidelines, or epidemiological tracking.
- *Virtual Healthcare Teams (VHT)*. It deals with healthcare professionals who collaborate and share information on patients through digital equipment.
- *Medical Research using Grids (MRuG)*. It means the powerful computing and data management capabilities to handle large amounts of heterogeneous data.

Table 2 shows the results of Search Types 1, 2, and 3 for Functionality. These results have been normalized in Table 3 which shows the final results that have been calculated considering an importance of 60% for Search Type 1, 30% for Search Type 2, and 10% for Search Type 3. The formulas are described as follows:

(1) tQinT1,i·0.60+nT2,i·0.30+nT3,i·0.10,

(2) nTj,i=vQj,i∑i=1nvQj,i,

where *t*_*Q*_*i*__ represents the final result that has been calculated considering each Search Type value for each Quality Characteristic  *i*: Search Type value 1 (*n*_*T*_1,*i*__), Search Type value 2 (*n*_*T*_2,*i*__), and Search Type value 3 (*n*_*T*_3,*i*__). For each Search Type value (*n*_*T*_*j*,*i*__), the formula is formula (2), where *v*_*Q*_*j*,*i*__ represents the quality characteristic value *i* for a Search Type *j*.

**Figure 2:**
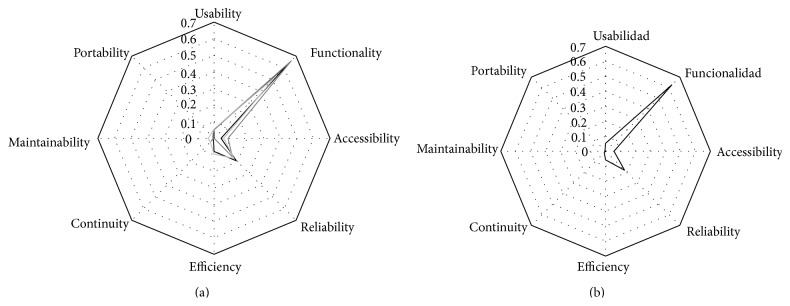
Internal/External Quality.

As far as results are concerned, the black, light grey, and grey lines in Figure 2(a) represent graphically the results of Search Types 1, 2, and 3, respectively. It can be observed that Functionality is much more studied than other Quality Characteristics such as Reliability, Efficiency, and Accessibility. There are very few studies on Usability, Continuity, Portability, and Maintainability.

The most studied Quality Characteristic by far is Functionality, as shown in Figure 2(b) which presents graphically the final results of Table 3. Quality Subcharacteristics of Functionality are being studied to a greater extent than others that may be more relevant for that matter. For instance, Safety (Quality in Use) is a significant Quality Characteristic, even though there are not as many studies on Safety as there are on Security or Interoperability (Quality Subcharacteristics of Functionality).

Most papers dealing with Trust describe users' ability to get confidence in e-Health Systems and they combine this concept with Reliability, which is defined as the ability of a system or component to perform its required functions under stated conditions for a specified period of time. In this sense, they are related because a Reliable system inspires users with Trust. Reliability is implicit in the standards implantation like Compliance, since such standards favor this Quality Characteristic. It is really troublesome to find papers based on availability (Quality Subcharacteristic of Reliability and Quality Characteristic in ITIL) of e-Health Systems. We guess that it undergoes the implicit idea that these systems have to be available 24/7. Nevertheless, techniques, processes, and systems responsible for this situation have to be defined in order to assure this service.  Table 4 shows the results of Search Types 1, 2, and 3 for Functionality. These results have been normalized in Table 5, which in turn shows the final results that have been calculated according to their importance, that is, 60% for Search Type 1, 30% for Search Type 2, and 10% for Search Type 3.

**Figure 3:**
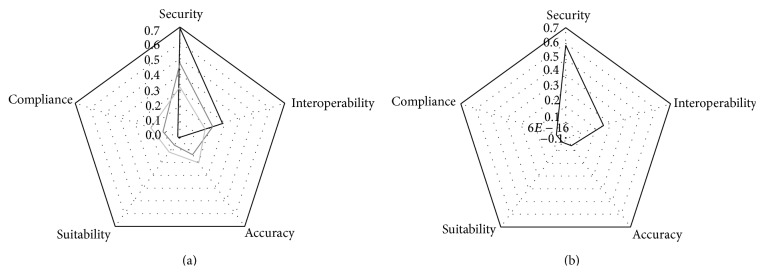
Quality subcharacteristics of functionality.

The black, light grey, and grey lines in Figure 3(a) represent graphically the results in Table 4 for Search Types 1, 2, and 3, respectively. It can be noticed that results differ in every type of search; however, Security and Interoperability are mainly in Search Types 1 and 2.

Accuracy, Compliance, and Suitability reach higher values than Interoperability, but lower than Security, in Search Type 3. This means that these words cope with a more general meaning of Quality in papers. Then, Security is the most studied Quality Characteristic of Functionality. As far as Security is concerned, most studies focus on Privacy and fewer papers exist dealing with Security focused on Integrity. Another aspect to take into account is the fact that Security is usually linked to Reliability in works dealing with quality. In regard to Interoperability, it must be mentioned that lots of works point out semantic interoperability.

Figure 3(b) shows graphically the results of Table 5. It represents that there are few studies about Compliance, Suitability, and Accuracy. Most papers about Functionality also deal with Security and Interoperability.

Table 6 shows the results of Search Types 1, 2, and 3 for Quality in Use. They have been normalized in Table 7. It outlines the final results that have been calculated considering an importance of 60% for Search Type 1, 30% for Search Type 2, and 10% for Search Type 3, respectively.

Actually, a shortage of Safety has been identified in e-Health Systems. It has to be noted that Safety should be a point of reference among all Quality Characteristics in e-Health Systems, but the fact still remains that academic people do not envisage this Quality Characteristic because there is lack of literature on this topic, in utter contrast to the literature available to other Quality Characteristics.

**Figure 4:**
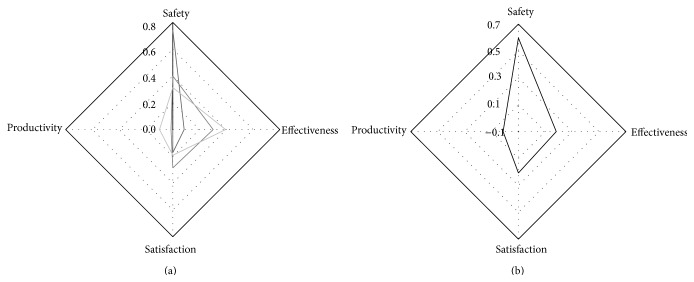
Quality in use.

The black, light grey, and grey lines in Figure 4(a) represent graphically the results of Table 7 for Search Types 1, 2, and 3, respectively. It can be observed that the proportion of Safety reaches higher rates in Search Types 1 and 2 than in Search Type 3. This means that Safety is a Quality Characteristic that has been studied regardless of other Quality Characteristics; that means Safety has been the topic of some work while Effectiveness and Satisfaction have been more generally applied in lots of papers.

Regarding Quality in Use, the Quality Characteristic of Effectiveness is the most used in e-Health Systems. Safety is the most studied Quality Characteristic, as represented in Figure 4(b) which shows graphically the final results considering the importance given to each Search Type. However, as mentioned before, Safety is less studied than any other Quality Characteristic which is given less importance. We would specially like to stress the relevance that this Quality Characteristic has, to the point of being essential in e-Health Systems. Productivity, Satisfaction, and Effectiveness, on their part, are not as much studied as Safety is.

## 3. A Proposed Framework to Manage e-Health Systems Quality

QuEF [9] is a framework that was initially developed for quality management of Model-Driven Web Development methodologies, but it has been extended to manage quality in other areas or domains like e-Health Systems. We guess that the use of QuEF would enhance e-Health Systems quality. QuEF can improve e-Health Systems efficiency in turn, making a more widespread use of those systems, since this evaluation helps anyone understand both the strengths and weaknesses of e-Health Systems. QuEF was actually extended in Eysenbach [1] with several phases:

- *Quality Model Strategy Phase*. It is the capital phase for the Quality Model life cycle concept and its main objective is turning the quality management into a strategic asses.
- *Quality Model Design Phase*. It gives advice on the Quality Model design, processes, and other aspects related to the Quality Model final design management effort. Significantly, design within QuEF is understood to encompass all relevant elements to plan the Quality Model.
- *Quality Model Transition Phase*. It helps execute changes in the Quality Model that have no influence on the operation phase. This phase covers how to manage these changes in the Quality Model.
- *Quality Model Operation Phase*. It recommends how to perform the analysis, evaluation, and planning of e-Health Systems quality continuous improvement. In this phase, the Quality Model is used to manage e-Health Systems quality.
- *Quality Model Continual Improvement Phase*. It aims to align and realign the Quality Model with the real needs to cover and the quality aspects to assure with the stakeholder of the approach. The Quality Model can change in terms of the identification of new trends or technological changes.

The phases above have been defined together with processes and artifacts to fulfill the complete Quality Model life cycle. The purpose of QuEF is to converge towards a continuous automatic quality improvement by means of generating Checklists and documentation as well as automatic evaluations and plans, so that quality can be controlled and implemented and, in turn, effort and time are automatically reduced.

## 4. Conclusions and Future Work

This paper lets people be aware of the mission and view of e-Health quality management and helps them understand how current studies evaluate e-Health Systems quality. This contribution focuses on detecting the most studied and used Quality Characteristics on e-Health Systems by means of a Systematic Literature Review. Therefore, a complete portfolio of Quality Characteristics to evaluate these Systems is proposed.

The objective of a future research is to reach a centralized and shared consensus on the Quality Model with clear goals agreed by all ICT organizations so as to implement e-Health Systems. This agreed Quality Model benefits the convergence towards a standardization and quality continuous improvement of e-Health Systems efficiently and effectively. In consequence, we propose QuEF, a framework to manage quality based on the Quality Model life cycle and define its extension for the e-Health Systems quality management. These Quality Characteristics are just part of the Quality Model.

In our view, the key is to give users what they really need (Properties) and, under Quality Characteristics, what they expect. Everything is embodied in the Quality Model, and ICT organizations know what they have to take into consideration. We guess that the use of QuEF would enhance e-Health Systems quality. Therefore, the QuEF can improve e-Health Systems efficiency in turn, making a more widespread use of those systems, since this evaluation helps anyone understand both the strengths and weaknesses of e-Health Systems.

The future and new goal will be twofold. On the one hand, we have to respond to questions such as the following: Have these Quality Characteristics been correctly studied? Are these Quality Characteristics aligned with those that users really demand? On the other hand, we must carry out a Systematic Literature Review for Properties to complete the Quality Model and characterize e-Health Systems, with the aim of knowing which are the most studied Properties in e-Health Systems and suggesting a set of Features of Properties for such purpose. Besides, another important aspect will be to define the importance of Properties and Quality Characteristics and to what extent the former has influence on the latter. Consequently, we will work on Group Decision Making process in order to reach a consensus on the Quality Model.

## Figures and Tables

**Table 1:** The review protocol.

Background	An e-Health System is figured out in the present work as a relatively recent term denoting healthcare practice supported by electronic processes and communication. However, it can also coin different meanings: some people argue that it is exchanged with Health informatics with a broader definition covering electronic/digital processes in Health, while others use it in the narrower sense of healthcare practice by means of the Internet. ICT organizations must guarantee stakeholders that a set of Quality Characteristics such as Usability, Functionality, or Maintainability must also be covered.

Research questions	What are the most studied and most used Quality Characteristics of e-Health Systems?

Strategy	Sources: Google Scholar, Scopus, Mendeley, Science Direct, ISI Web of Knowledge, ACM Digital Library, CiteSeerX, or the IEEE digital library. Keywords: “Quality” AND “e-Health”, “Evaluation” AND “e-Health”, “Assessment” AND “e-Health”, “Technology” AND “Health”, or “Communication” AND “Health”.

Study selection criteria	All kinds of articles (conferences and journal articles, patents, websites, conference proceedings, doctoral dissertations, Open Access material, and some others) related to the quality evaluation of e-Health Systems, published from 2006. Three search types of logical criterion in the search field: (i) Search Type 1: Title field, (ii) Search Type 2: Title field, Abstract field, and Keywords field, (iii) Search Type 3: All fields.

Study selection procedures	The article contains any kind of evaluation or assessment about any e-Health Systems.

Data extraction strategy	The data extracted from each paper will be as follows: Source (i.e., the conference or journal), year when the paper was published, classification of the paper (type and scope, research trends, or specific research question), topic area, author(s), summary of the paper, and quality score for the study.

**Table 2:** Number of results of Search Types 1, 2, and 3, respectively, for Quality Characteristics.

	Usability	Functionality	Accessibility	Reliability
Search Type 1	11	134	9	41
Search Type 2	59	723	93	173
Search Type 3	2.114	19.788	2.213	6.515

	Efficiency	Continuity	Maintainability	Portability

Search Type 1	17	1	0	0
Search Type 2	0	28	5	12
Search Type 3	3.623	1.177	1.308	1.115

**Table 3:** Normalized value of the results obtained in Search Types 1, 2, and 3, respectively, for Quality Characteristics.

Importance	Usability	Functionality	Accessibility	Reliability
60%	0.05164319	0.62910798	0.04225352	0.19248826
30%	0.05397987	0.66148216	0.08508692	0.15827996
10%	0.05585594	0.52275449	0.05846249	0.17211166
	**0.05276547**	**0.62818489**	**0.05672444**	**0.18018811**

Importance	Efficiency	Continuity	Maintainability	Portability

60%	0.07981221	0.00469484	0	0
30%	0.00000000	0.02561757	0.00457457	0.01097896
10%	0.09570271	0.03110250	0.03455442	0.02945579
	**0.0574576**	**0.01361242**	**0.00482781**	**0.00623927**

**Table 4:** Number of results of Search Types 1, 2, and 3, respectively, for Functionality.

	Security	Interoperability	Accuracy	Suitability	Compliance
Search Type 1	93	38	0	1	2
Search Type 2	340	158	99	44	82
Search Type 3	6.328	3.330	3.994	2.302	3.834

**Table 5:** Normalized value of the results of Search Types 1, 2, and 3, respectively, for Functionality.

Importance	Security	Interoperability	Accuracy	Suitability	Compliance
60%	0.69402985	0.28358209	0.00000000	0.00746269	0.01492537
30%	0.47026279	0.21853389	0.13692946	0.06085754	0.11341632
10%	0.31980662	0.16828381	0.20183950	0.11631629	0.19375379
	**0.58947741**	**0.2525378**	**0.06126279**	**0.0343665**	**0.0623555**

**Table 6:** Number of results of Search Types 1, 2, and 3, respectively, for Quality in Use.

	Safety	Effectiveness	Satisfaction	Productivity
Search Type 1	26	3	6	0
Search Type 2	127	96	91	4
Search Type 3	4.909	6.172	2.997	1.554

**Table 7:** Normalized value of the results of Search Types 1, 2, and 3, respectively, for Functionality.

Importance	Safety	Effectiveness	Satisfaction	Productivity
60%	0.74285714	0.08571429	0.17142857	0.00000000
30%	0.39937107	0.30188679	0.28616352	0.01257862
10%	0.31402738	0.39482663	0.19170896	0.09943703
	**0.59692834**	**0.18147727**	**0.2078771**	**0.01371729**
